# What makes us more susceptible to false memories in the era of COVID‐19? A focus on vaccines and Green Pass

**DOI:** 10.1002/brb3.2815

**Published:** 2022-11-30

**Authors:** Chiara Scuotto, Ciro Rosario Ilardi, Gianpaolo Maggi, Alfonso Ilardi, Nadia Gamboz, Maria Staiano, Giovanni Borrelli, Marco La Marra, Raffaella Perrella

**Affiliations:** ^1^ Department of Psychology University of Campania Luigi Vanvitelli Caserta Italy; ^2^ Inmates Ward, Department of Internal Medicine Antonio Cardarelli Hospital Naples Italy; ^3^ Laboratory of Experimental Psychology Suor Orsola Benincasa University Naples Italy; ^4^ Department of Experimental Medicine University of Campania Luigi Vanvitelli Naples Italy

**Keywords:** attitude, COVID‐19, fake news, false memories

## Abstract

**Introduction:**

The coronavirus disease 2019 (COVID‐19) pandemic was accompanied by an overabundance of fake news increasing the risk of developing false memories (FMs). Previous studies have shown that the relationship between fake news and FMs could be mediated by some individual variables, including attitudinal biases. We explored the role of these variables in true memories (TMs) and FMs formation, with special emphasis on vaccine‐ and Green Pass (GP)‐related topics.

**Method:**

We set up a large online survey exploring several constructs including media usage, attitude toward vaccines and GP, perceived (PK) and objective knowledge (OK) about COVID‐19‐related information, fear of the disease, depression and anxiety symptoms, coping mechanisms, and reasoning skills. Then, we asked participants whether they remembered certain news (true or fake), providing confidence ratings.

**Results:**

Data from 289 respondents (198 females) from the general population were analyzed. Participants with positive attitude reported a greater fear that their loved ones contracted the COVID‐19, a more frequent use of traditional media, and a higher PK when compared with respondents with negative attitude. On the whole sample, participants reported higher confidence levels when required to judge their memory of true than fake news; however, participants with positive attitude reported a higher confidence for both true and fake news. The relationship between attitude and TM confidence was mediated by the PK, whereas the relationship between attitude and FM confidence was probably affected by OK.

**Conclusion:**

Attitude can modulate individual behaviors in the context of health issues. The PK and OK may interact with attitude in the memory formation.

## Graphical abstract

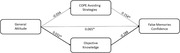

## 1 INTRODUCTION

False memories (FMs) refer to the recollection of events that never happened and/or are remembered differently from how they actually occurred (Roediger & McDermott, 1995). Previous research has demonstrated that several individual variables can affect the development of FMs.

It has been reported that FMs emerge mainly in individuals showing a strong interest/involvement in a given topic (O'Connel & Greene, 2017). In other words, these individuals display a high perceived knowledge (PK, Mehta et al., 2011) leading to overestimate their memory abilities. People showing high PK tend to remember any kind of information, whether it is true or false, because they are reluctant to admit ignorance about the topic of their interest (Mehta et al., 2011). Conversely, objective knowledge (OK, Frenda et al., 2013) promotes the development of complex cognitive reasoning patterns on a given topic and appears to be associated with a lower frequency of FMs (Frenda et al., 2013; Greene & Murphy, 2020).

Other variables that may affect the production of FMs are the information sources (Scuotto et al., 2021), symptoms of anxiety and depression (Storbeck et al., 2005; Zhu et al., 2010), and analytical skills (e.g., logical reasoning and abstraction abilities; Pennycook et al., 2019; Zhu et al., 2010). In this vein, it has been proposed that impoverishment of executive/frontal functions (Ilardi, Chieffi, et al., 2022; Ilardi, Iavarone, et al., 2022; La Marra, Ilardi, et al., 2022; La Marra, Villano, et al., 2022), implicating a disconnection between source monitoring and recall strategies, as well as a decreased effectiveness of updating and inhibition processes, is associated with FMs creation (Devitt & Schacter, 2016; Plancher et al., 2009). In addition, the production of FMs can be modulated by emotions. Indeed, it seems that people are more alert, and pay more attention, to relevant environmental stimuli (Chieffi et al., 2019, 2014; Knight et al., 2007) when they experience negative emotions (Kaplan et al., 2015). Finally, coping strategies based on problem‐solving, help‐seeking, and avoidance behaviors were found to be associated with the formation of FMs, particularly in stressful situations (Babore et al., 2020; Gudjonsson, 1988; Rettie & Daniels, 2021).

When a major social event occurs, there is typically a massive proliferation of media reports. This often makes information ambiguous and/or difficult to interpret. In fact, the news that are disseminated may be either misinterpreted by the users (Lazer et al., 2018; Scuotto et al., 2021) or purposely distorted by the source, that i, fake news (Tandoc, 2019). In this respect, previous studies have suggested that exposure to fake news can also lead to the development of FMs (Awan et al., 2022; Greene & Murphy, 2020; Greene et al., 2022; Scuotto et al., 2021; Zhu et al., 2010).

The coronavirus disease 2019 (COVID‐19) pandemic was accompanied by an “infodemic,” that is, an overabundance of confusing information conveying potentially harmful messages and/or promoting inadequate behaviors (Apuke & Omar, 2021). Studying the dissemination of misinformation and fake news is particularly relevant in the current scenario. Indeed, the copious quantity of fake news spread during the COVID‐19 pandemic could have facilitated the emergence of FMs that, in turn, represent significant predictors of mental health issues, cognitive failures (Maggi et al., 2021, 2022; Santangelo et al., 2021), and lack of implementation of preventive health behaviors (Greene & Murphy, 2020; Nash et al., 2016). Notable in this vein are the adherence to anti‐SARS‐CoV‐2 vaccination campaign and, as happens in Italy, the Green Pass (GP) release (Catalan‐Matamoros et al., 2020; Kim & Kim, 2020). For the sake of clarity, the GP is a certificate showing that people have been vaccinated, tested negative, or recovered from COVID‐19.

In a previous study by our research group (Scuotto et al., 2021), the predictive value of different individual/personological variables (i.e., use of traditional and social media, PK and OK, fear of the disease, depression and anxiety symptoms, reasoning skills, and coping mechanisms) on the production of FMs for COVID‐19‐related news was tested in a sample of Italian university students. The current study represents an extension of the previous one, overcoming some of its limitations. In this regard, besides the weak external validity, we worked only on raw recollection scores (i.e., “I remember the news” = 1 point vs. “I don't remember the news” = 0 point) without asking participants to indicate the level of confidence about their memory and source memory (Brainerd & Reyna, 2002; Kim & Cabeza, 2007). In the case of a true or false recollection, merely analyzing raw dichotomic scores may lead to inflating the estimate of memory accuracy as responses may be affected by memory biases. Conversely, working on confidence ratings enables memory judgments to be properly weighed, gaining in terms of measurement validity.

Furthermore, as recently proposed, the role of attitudinal bias should be taken into account when investigating the susceptibility to FMs (Hegarty et al., 2021). The attitude toward a given “object” conveys a whole assessment encompassing cognitive, emotional, and behavioral responses that can affect the encoding of object‐related information (Rosenberg & Hovland, 1960). Thus, it is expected that the attitude valence (positive or negative) could be critical in the development of FMs. In line with this claim, here we focused on the relationship between attitude toward vaccines and GP and memories for vaccines‐ and GP‐related news.

## 2 METHODS

### 2.1 Participants

Three hundred and ten subjects (212 females) participated in the study. Most of them (68.67%) lived in the Campania region (Southern Italy). Mean age of participants was 34.36 years (SD = 14.31), whereas mean education was 15.80 years (SD = 2.34). Inclusion criteria were age ≥18 years, education ≥5 years (i.e., primary school), completion of at least the first dose of anti‐SARS‐CoV‐2 vaccine and obtaining the GP. Exclusion criteria were the history of neurocognitive, psychiatric, or psychopathological disorders, serious health conditions including cancer, diabetes, and severe obesity, and alcohol or substance abuse/addiction. Health conditions within the exclusion criteria were disclosed when informed consent was granted.

### 2.2 Materials and procedure

Participants filled a survey (about 40 min to complete) through the Google Forms platform. The survey was disseminated via the web by word of mouth (e.g., family members, friends, colleagues, university students) and social networks (e.g., Facebook, Instagram, WhatsApp). It consisted of six sections and, in order to avoid treating missing data, we set up the platform so that it was mandatory to answer each question.

#### 2.2.1 Sociodemographic data

Participants were briefly informed about the study's aims and digitally gave their consent to participate. Thereafter, they generated an identification code (i.e., the first letter of the first name, the first letter of the last name, and birth date) and provided socio‐anagraphic data (i.e., sex, age, education, profession, and region of origin).

#### 2.2.2 COVID‐19‐related information

Participants were asked whether they (i) had contracted the Coronavirus infection, (ii) were vaccinated, (iii) held the GP, and (iv) when they were vaccinated for the first time (January–March, 2021; April–June, 2021; July–October, 2021; November, 2021–March, 2022). Participants were finally requested to judge, on a 5‐point Likert scale, how afraid they were of contracting, and that their loved ones contracting, the virus (0 = *not at all*, 4 = *a lot*).

#### 2.2.3 Media usage

Participants rated the frequency of the use of traditional (e.g., television) and social media (e.g., Facebook, Twitter, Instagram, LinkedIn, YouTube, TikTok) to gain information about the course of the COVID‐19 pandemic (0 = *almost never*, 4 = *very often*). Moreover, we asked participants to rate, on the same Likert scale, the frequency of use of specific information sources for both media, that is, newspaper articles, radio, and television for traditional media, and channels/profiles/pages edited by political figures, show business personalities, official radio newspapers, health professionals, scientific publishing, and family/friends for social media.

#### 2.2.4 Attitude toward vaccines and Green Pass, perceived knowledge, and objective knowledge

Three ad hoc–devised questionnaires were presented (see Appendix A). The first one (8 items) was constructed to explore the attitude toward anti‐SARS‐CoV‐2 vaccines and GP by expressing the degree of agreement (Likert scale 0−4, 0 = *strongly disagree*, 4 = *strongly agree*) with respect to certain statements concerning vaccines and GP (e.g., *The vaccine is safe*, *The Green Pass is a tool that protects personal freedoms*). For the sake of brevity, attitude toward vaccines and GP will be referred, now onwards, merely as “attitude.” The second one (9 items) was devised to assess the PK about COVID‐19 (Likert scale 0−4, 0 = *no knowledge*, 4 = *excellent knowledge*), that is, how competent participants considered themselves to be about the topics “COVID‐19” (in general), vaccinations, and GP. Lastly, the third one was a true/false quiz (12 statements, 6 true and 6 false) used to measure the OK about COVID‐19, with a focus on the vaccine and GP topics. To compute the OK score, one point was given to each statement correctly recognized as true or false.

#### 2.2.5 Depression, anxiety, coping mechanisms, and logical reasoning

Levels of depression and anxiety were assessed by using the Beck Depression Inventory (BDI; Beck et al., 1961) and the Trait scale of the State‐Trait Anxiety Inventory‐Form Y (T‐Anxiety‐STAI‐Y; Ilardi, Gamboz, et al., 2021), respectively. Coping mechanisms were measured through four out of the five subscales of the COPE‐New Italian Version‐25 (COPE‐NVI‐25; Foà et al., 2015), that is, COPE‐avoidance strategies, COPE‐positive attitude, COPE‐social support, and COPE‐problem‐solving. The cognitive estimation task (CET; Della Sala et al., 2003) was used as a measure of reasoning and self‐monitoring skills, contemplated within the frontal/executive functioning.

#### 2.2.6 Misinformation‐based experimental paradigm

Subjects were presented with 12 vaccine‐ and GP‐related news, 6 of which were true and 6 were fake (see Appendix B). Fake news were totally fabricated and constructed after a careful examination of possible fake news already spread on the web and/or television. Therefore, reporting news items to which the subjects could have actually been exposed was prevented. True news were instead extrapolated from the websites of the World Health Organization and the Italian Ministry of Health. It is important to underline that the use of “new” fake news rather than “old” fake news to which participants could have been exposed was essential to test the presence of FMs. Indeed, the recollection of an already known fake news should be classified, logically speaking, as a true memory (TM). Conversely, the recollection of a newly constructed fake news is to be considered an effective FM because the subject should not be able to access any memory trace embracing the information at hand. The experimental procedure was as follows. Participants were asked whether they remembered (*I remember this*) or not (*I do not remember this*) each news. If participants reported remembering news, they had to provide a conference rating on their memory by using a 5‐point Likert scale (1 = *Not much confident*, 5 = *Very much confident*). They were asked to provide a confidence rating also about the possible source memory, that is, traditional and social media, using the same response set. The dependent measures were confidence scores obtained by summing separately the confidence ratings for true (TMC) and fake news (FMC). Specifically, if a subject mistakenly recalls—for instance—3/6 fake news, giving each a confidence rating of 2, 2, and 3, respectively, her/his FMC score will be equal to 7. TMC‐ and FMC‐traditional media and TMC‐ and FMC‐social media will be the labels used to indicate confidence levels concerning the media source.

This research project was designed and conducted according to the ethical standards as laid down in the 1964 Declaration of Helsinki and its later amendments and approved by the Ethics Committee of the University of Campania “Luigi Vanvitelli” (protocol code: 3/2022, date of approval by the Department of Psychology: 25 January 2022).

### 2.3 Statistical analyses

Descriptive statistics (frequency for nominal variables; mean and standard deviation for continuous variables) were reported for the whole sample. Response rates and confidence levels for each true and fake news were analyzed by one‐way *χ* ^2^ (Scuotto et al., 2021) and repeated‐measures analysis of variance (ANOVA) with Greenhouse–Geisser correction, as applicable.

The median value of the attitude score was used to split participants into two subgroups (negative vs. positive attitude) that were contrasted on the variables of interest (i.e., sociodemographic data, COVID‐19‐related information, use of traditional and social media, PK score, OK score, BDI score, T‐anxiety score, COPE subscales, CET score, TMC score, FMC score, and related confidence levels for source memory) via one‐ or two‐way chi‐squared tests (*χ* ^2^) and univariate ANOVA, as appropriate. To further investigate the relationships between attitude and confidence ratings (TMC vs. FMC, TMC‐traditional media vs. TMC‐social media, FMC‐traditional media vs. FMC‐social media), mixed factorial ANOVA was used.

Pearson's correlation (*r*) analysis was run in order to select potential mediators to load in the mediation analysis. Indeed, mediation models were constructed for testing whether the relationship between attitude and memories was subtended by other explanatory variables. To evaluate the significance of direct and indirect effects, bootstrapping procedure with 5000 samples with replacement from the full sample to construct bias‐corrected 95% confidence intervals was conducted by SPSS Macro PROCESS (Scuotto et al., 2021). Overall, a *p*‐value <.05 was considered statistically significant. Effect sizes were computed by means of Phi coefficient (*φ*), Cramér's V (Ilardi et al., 2020), eta (*η* ^2^), or partial eta squared ($\eta_{p}^{2}$). All statistical analyses were performed by IBM SPSS Statistics version 26.0 and JASP version 0.16.

## 3 RESULTS

### 3.1 Sample characteristics

Out of a total of 310 respondents, 9 were excluded as they had not been vaccinated and/or lacked the GP. Data from 12 subjects were removed since they got BDI, T‐Anxiety, and/or CET *z*‐scores equal to, or greater than, 2.6 in absolute terms (i.e., univariate outliers). Therefore, the final sample included 289 participants (198 females; M age = 34.46, SD = 13.54, age range = 18−74 years; M education = 15.82, SD = 2.36; education range = 8−18 years), of which 152 exhibited negative attitude (M = 16.01, SD = 5.86) and 137 exhibited positive attitude (M = 27.80, SD = 2.53). The median value of the attitude score (M = 21.60, SD = 7.46) was 23. Descriptive statistics for each section of the survey are reported in Table 1. Results of the comparisons between participants with positive and negative attitudes are summarized in Table 1 and detailed in the following sections.

**TABLE 1:** Descriptive statistics

	Variables	Total sample	Negative attitude	Positive attitude	*p*	Effect size
	*N*	289	152	137		
Section 1	Age in years^a^	34.46 ± 13.54	33.78 ± 12.57	35.23 ± 14.54	NS	
	Educational level in years^a^	15.82 ± 2.36	14.54 ± 2.51	16.14 ± 2.13	*	0.02^e^
	Sex (F/M)	198/91	112/40	86/51	*	0.12^c^
	Location					
	Campania^b^	207 (71.6)	119 (78.3)	88 (64.2)	–	
	Lazio^b^	11 (3.8)	5 (3.3)	6 (4.4)	–	
	Lombardia^b^	18 (6.2)	9 (5.9)	9 (6.6)	–	
	Sicilia^b^	26 (9.0)	9 (5.9)	17 (12.4)	–	
	Other Italian regions^b^	27 (9.4)	10 (6.6)	17 (12.4)	–	
	Profession					
	Employer^b^	49 (17.0)	27 (17.8)	22 (16.1)	NS	
	Freelancer^b^	28 (9.7)	15 (9.9)	13 (9.5)	NS	
	Physician^b^	27 (9.3)	7 (4.6)	20 (14.6)	*	0.48^d^
	Psychologist^b^	28 (9.7)	15 (9.9)	13 (9.5)	NS	
	Teacher^b^	44 (15.2)	24 (15.8)	20 (14.6)	NS	
	University student^b^	78 (27.0)	43 (28.3)	35 (25.5)	NS	
	Other^b^	35 (12.1)	21 (13.8)	14 (10.2)	–	
Section 2	First anti‐SARS‐CoV‐2 vaccination					
	Between January and March 2021^b^	86 (29.8)	35 (23.0)	51 (37.2)	NS	
	Between April and June 2021^b^	98 (33.9)	46 (30.3)	52 (38.0)	NS	
	Between July and October 2021^b^	93 (32.2)	60 (39.5)	33 (24.1)	**	0.29^d^
	Between November 2021 and March 2022^b^	12 (4.2)	11 (7.2)	1 (0.7)	**	0.83^d^
	*n* contracting the COVID‐19^b^	88 (30.4)	55 (36.2)	33 (24.1)	*	0.25^d^
	Fear of contracting COVID‐19^a^	2.27 ± 1.18	2.21 ± 1.26	2.33 ± 1.09	NS	
	Fear that loved ones contracting COVID‐19^a^	3.22 ± 0.94	3.09 ± 1.06	3.36 ± 0.78	*	0.02^e^
Section 3	Use of traditional media^a^	3.11 ± 0.99	3.00 ± 1.09	3.24 ± 0.86	*	0.01^e^
	Use of social media^a^	2.59 ± 1.38	2.78 ± 1.27	2.38 ± 1.46	*	0.02^e^
Section 4	Attitude^a^	21.60 ± 7.46	16.01 ± 5.86	27.80 ± 2.53	–	
	PK score^a^	23.92 ± 5.99	22.15 ± 5.79	25.88 ± 5.61	***	0.10^e^
	OK score^a^	8.58 ± 1.52	8.46 ± 1.65	8.72 ± 1.35	NS	
Section 5	BDI score^a^	11.82 ± 7.52	12.53 ± 7.71	11.02 ± 7.24	NS	
	T‐Anxiety score (STAI‐Y)^a^	46.86 ± 11.90	48.01 ± 11.81	45.57 ± 11.92	NS	
	COPE‐avoidance strategies^a^	12.66 ± 4.60	12.80 ± 4.43	12.50 ± 4.78	NS	
	COPE‐positive attitude^a^	26.26 ± 4.86	25.96 ± 4.67	26.59 ± 5.05	NS	
	COPE‐social support^a^	18.89 ± 5.64	18.83 ± 5.62	18.95 ± 5.68	NS	
	COPE‐problem solving^a^	21.74 ± 3.93	21.44 ± 3.49	22.07 ± 4.36	NS	
	CET score^a^	12.71 ± 5.01	12.80 ± 5.15	12.61 ± 4.87	NS	
Section 6	TMC score^a^	9.35 ± 5.34	8.45 ± 5.11	10.14 ± 5.48	**	0.02^e^
	FMC score^a^	2.99 ± 3.81	2.55 ± 3.17	3.47 ± 4.38	*	0.01^e^
	TMC‐traditional media^a^	9.31 ± 5.50	8.40 ± 4.96	10.32 ± 5.89	**	0.03^e^
	TMC‐social media^a^	6.24 ± 4.56	6.05 ± 4.11	6.45 ± 5.01	NS	
	FMC‐traditional media^a^	2.91 ± 3.74	2.50 ± 3.07	3.36 ± 4.33	*	0.01^e^
	FMC‐social media^a^	2.15 ± 3.16	1.82 ± 2.52	2.52 ± 3.71	NS	

Abbreviations: BDI, Beck Depression Inventory; CET, cognitive estimation task; COPE, COPE—New Italian Version‐25; COVID‐19, coronavirus disease 2019; FMC, false memories confidence; OK, objective knowledge; PK, perceived knowledge; STAI, State‐Trait Anxiety Inventory; TMC, true memories confidence.

^a^ Mean ± SD.

^b^ Frequency (%).

^c^ Phi coefficient.

^d^ Cramér's V.

^e^ Eta squared.

*p<.05, **p<.01, ***p<.001

### 3.2 Sociodemographic data

Women reported a more negative attitude as compared with men (*χ* ^2^(1) = 3.976, *p* = .046). Furthermore, participants with positive attitude were more educated than those with negative attitude (*F*(1, 287) = 4.705, *p* = .031). No between‐group differences emerged in terms of age.

### 3.3 COVID‐19‐related information

We found differences between participants with negative and positive attitudes on the date of the first anti‐SARS‐CoV‐2 vaccination, with a larger number of respondents with negative attitude that have undergone vaccination in the latest time frames, that is, July–October 2021 (*χ* ^2^(1) = 7.839, *p* = .005) and November 2021–March 2022 (*χ* ^2^(1) = 8.333, *p* = .004). Moreover, participants with negative attitude reported having been infected more than those with positive attitude (*χ* ^2^(1) = 5.500, *p* = .019). Respondents with positive attitude reported more fear that their loved ones contracting the COVID‐19 (*F*(1, 287) = 6.366, *p* = .012) than respondents with negative attitude.

### 3.4 Media usage

Participants with positive attitude reported a more frequent use of traditional media for acquiring information regarding COVID‐19 (*F*(1, 287) = 4.239, *p* = .040), whereas individuals with negative attitude made greater use of social media (*F*(1, 287) = 6.252, *p* = .013). Interestingly, results from sub‐analyses revealed that participants with positive attitude consulted more often social media channels of scientific publishing (*F*(1, 287) = 4.962, *p* = .027, *η* ^2^ = .017), whereas participants with negative attitude those of celebrities (*F*(1, 287) = 7.783, *p* = .030, *η* ^2^ = .016) and family/friends (*F*(1, 287) = 7.490, *p* = .007, *η* ^2^ = .025).

### 3.5 Perceived and objective knowledge

Respondents with positive attitude showed higher PK (*F*(1, 287) = 30.655, *p* < .001) than respondents with negative attitude. Nevertheless, no difference was detected on the OK score.

### 3.6 Depression, anxiety, coping mechanisms, and logical reasoning

No significant differences were found between participants with positive and negative attitudes on the BDI, T‐Anxiety, and CET scores, or even on the four COPE subscales (all *p*_s_ > .09).

### 3.7 True and false memories

Response rates and confidence ratings for each true and fake news are reported in Table 2. True news were labeled as TN1, TN2, TN3, TN4, TN5, and TN6, whereas fake news as FN1, FN2, FN3, FN4, FN5, and FN6 (see Appendix B). As concerns true news, a large proportion of participants recalled TN1 (*χ* ^2^(1) = 20.516, *p* < .001, *φ* = .266) and TN6 (*χ* ^2^(1) = 76.820, *p* < .001, *φ* = .515); TN2 was recalled by about half of participants (*χ* ^2^(1) = 2.522, *p* = .112); the majority of participants, instead, did not remember TN3 (*χ* ^2^(1) = 54.066, *p* < .001, *φ* = .432), TN4 (*χ* ^2^(1) = 113.360, *p* < .001, *φ* = .626), and TN5 (*χ* ^2^(1) = 78.896, *p* < .001, *φ* = .522). As for confidence ratings for correctly remembered true news (TMC), a significant difference emerged from repeated‐measures ANOVA (*F*(5, 1347) = 91.976, *p* < .001, $\eta_{p}^{2}$ = .242) according to the Greenhouse–Geisser correction. In sum, the pairwise comparisons showed that participants who correctly recalled the true news were more confident for TN1, TN2, and TN6; particularly, TN6 was recalled with higher confidence overall. Finally, for all TMs, source memory (TMC‐traditional media vs. TMC‐social media) was ascribed more confidently to traditional than social media (all repeated‐ANOVAs were statistically significant for *p* < .01, $\eta_{p}^{2}$ range = .031−.247).

**TABLE 2:** Response rates and confidence ratings for true and fake news

True news	TN1	TN2	TN3	TN4	TN5	TN6
Recalled, frequency (%)	183 (63.3)	131 (45.3)	82 (28.4)	54 (18.7)	69 (23.9)	219 (75.8)
TMC, mean ± SD	2.07 ± 1.78	1.67 ± 1.96	0.99 ± 1.67	0.54 ± 1.21	0.94 ± 1.74	3.04 ± 1.90
TMC‐traditional media, mean ± SD	2.37 ± 1.99	1.50 ± 1.86	1.00 ± 1.71	0.62 ± 1.41	0.91 ± 1.72	2.91 ± 1.92
TMC‐social media, mean ± SD	1.53 ± 1.58	1.06 ± 1.49	0.53 ± 1.04	0.46 ± 1.13	0.61 ± 1.27	2.05 (1.70)
Fake news	FN1	FN2	FN3	FN4	FN5	FN6
Recalled, frequency (%)	40 (13.8)	12 (4.2)	30 (10.4)	32 (11.1)	15 (5.2)	107 (37.0)
FMC, mean ± SD	0.44 ± 1.18	0.15 ± 0.75	0.34 ± 1.05	0.99 ± 1.67	0.18 ± 0.78	1.46 ± 1.98
FMC‐traditional media, mean ± SD	0.45 ± 1.19	0.16 ± 0.77	0.36 ± 1.10	1.00 ± 1.71	0.17 ± 0.78	1.34 ± 1.89
FMC‐social media, mean ± SD	0.36 ± 0.99	0.12 ± 0.65	0.25 ± 0.79	0.53 ± 1.04	0.13 ± 0.63	1.01 ± 1.57

*Note*: For news’ acronyms, see Appendix B.

Abbreviations: FMC, false memories confidence; TMC, true memories confidence.

About fake news, most of the respondents did not remember seeing/hearing them (all one‐way *χ* ^2^ tested were statistically significant for *p* < .001, *φ* range = .259−.916). A significant difference emerged on confidence ratings (FMC) as highlighted by repeated‐measures ANOVA (*F*(3, 1025) = 42.917, *p* < .001, $\eta_{p}^{2}$ = .13) with the Greenhouse–Geisser correction. According to pairwise comparisons, participants who recalled the fake news showed the highest confidence for their memory of FN6. As was the case for the true news, source memory for each fake news (FMC‐traditional media vs. FMC‐social media) was attributed more confidently to traditional than social media (all repeated‐ANOVAs were statistically significant for *p* < .05, $\eta_{p}^{2}$ range = .014−.082).

### 3.8 Memories and memory source in participants with positive and negative attitudes

As concerns attitude‐related differences, participants with positive attitude were more confident about their memory for both true (TMC, *F*(1, 287) = 7.365, *p* = .007) and fake news (FMC, *F*(1, 287) = 4.180, *p* = .042) and reported higher levels of confidence in judging traditional media as source of information for both TMs (TMC‐traditional media, *F*(1, 287) = 9.028, *p* = .003) and FMs (FMC‐traditional media, *F*(1, 287) = 3.886, *p* = .049) than participants with negative attitude. No difference was found on confidence ratings regarding social media as the source of TMs (TMC‐social media, *F*(1, 287) = .535, *p* = .465), whereas a trend toward significance emerged by comparing the two groups on confidence levels about social media as source of FMs (FMC‐social media, *F*(1, 287) = 3.529, *p* = .061). Specifically, respondents with positive attitude reported slightly higher confidence levels than respondents with negative attitude.

**FIGURE 1:**
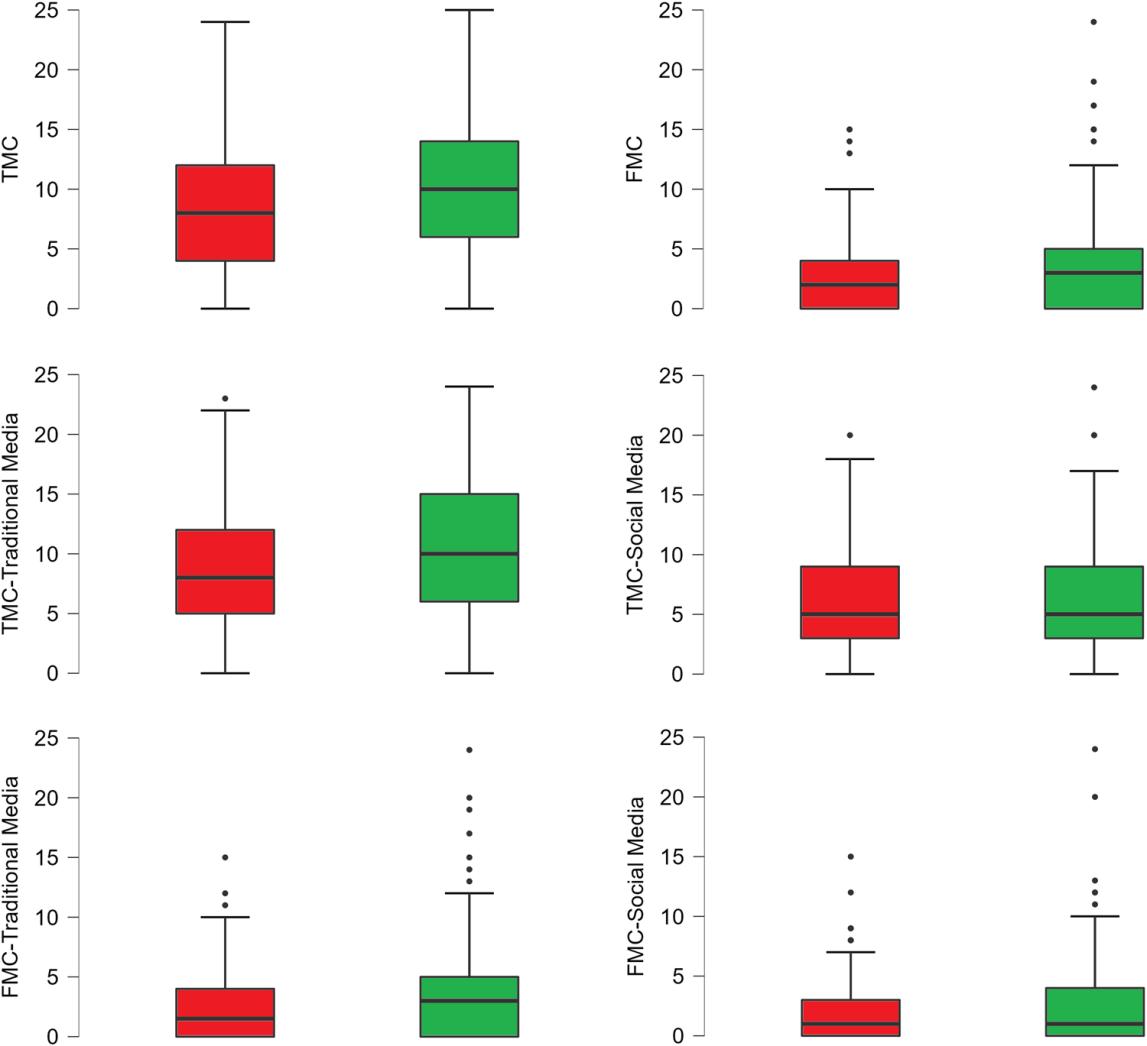
Boxplots depicting differences between participants with negative (red boxes) and positive (green boxes) attitudes on true memory confidence (TMC) and false memory confidence (FMC) scores

A more fine‐grained analysis was performed to compare confidence ratings among respondents with positive and negative attitudes. We ran three 2 × 2 mixed factorial ANOVAs, where the group (positive vs. negative attitude) was the between‐subjects factor, and the confidence rating (model 1 = TMC vs. FMC; model 2 = TMC‐traditional media vs. TMC‐social media; model 3 = FMC‐traditional media vs. FMC‐social media) was the within‐subjects factor (see Figure 1).

In the first model, main effects of confidence rating (*F*(1, 287) = 405.133, *p* < .001, $\eta_{p}^{2}$ = .585) and group (*F*(1, 287) = 8.626, *p* = .004, $\eta_{p}^{2}$ = .029) were detected. Particularly, participants were generally more confident in remembering true than fake news (mean diff: 6.283, SE = 0.312, *t* = 20.128, *p* < .001), although respondents with positive attitude were more confident of their memories of both true and fake news (see Table 1). No group × confidence interaction (*F*(1, 287) = 1.548, *p* = .214) emerged from the analysis.

The second model revealed a main effect of confidence rating (*F*(1, 287) = 126.552, *p* < .001, $\eta_{p}^{2}$ = .306), a main group effect (*F*(1, 287) = 8.626, *p* = .004, $\eta_{p}^{2}$ = .029), and a significant group × confidence interaction (*F*(1, 287) = 4.916, *p* = .027, $\eta_{p}^{2}$ = .017). Specifically, participants who correctly remembered the true news were more confident in judging traditional media as a source of their memories as compared with social media (mean diff: 3.112, SE = 0.277, *t* = 11.250, *p* < .001); additionally, participants with positive attitude reported higher levels of confidence as compared with participants with negative attitude if data are averaged over both TMC‐media levels (mean diff: 1.157, SE = 0.522, *t* = 2.217, *p* = .027). However, considering separately each within‐subject factor level, they reported higher confidence ratings for traditional (mean diff: 1.921, SE = 0.591, *t* = 3.252, *p* = .007) but not for social media (mean diff: 0.393, SE = 0.591, *t* = .666, *p* = .999).

The third model showed significant main effects of confidence (*F*(1, 287) = 45.629, *p* < .001, $\eta_{p}^{2}$ = .137) and group (*F*(1, 287) = 3.997, *p* = .047, $\eta_{p}^{2}$ = .014) but no interaction between the two factors (*F*(1, 287) = .567, *p* = .452). On average, respondents reported higher confidence for traditional media when required to judge the source of their FMs (mean diff: 0.755, SE = 0.112, *t* = 6.755, *p* < .001); however, when data are averaged over both FMC‐traditional and FMC‐social media, participants with positive attitude reported higher confidence levels for both traditional and social media as compared with those showing negative attitude (mean diff: 0.781, SE = 0.391, *t* = 1.999, *p* < .047). This result is in line with the trend toward significance highlighted by univariate comparisons (see Table 1).

### 3.9 Correlation analysis

Correlation analyses showed that TMC score was significantly associated with age (*r* = .243, *p* < .001), PK score (*r* = .267, *p* < .001), attitude (*r* = .200, *p* = .001), fear that loved ones contracting COVID‐19 (*r* = .119, *p* = .044), T‐Anxiety score (*r* = −.159, *p* = .007), COPE‐positive attitude (*r* = .245, *p* < .001), and COPE‐problem‐solving (*r* = .166, *p* = .005) subscales. Otherwise, FMC score was significantly related to age (*r* = .120, *p* = .042), educational level (*r* = −.123, *p* = .037), OK score (*r* = −.127, *p* = .031), fear of contracting COVID‐19 personally (*r* = .122, *p* = .039), CET score (*r* = .118, *p* = .045), and COPE‐avoiding strategies subscale (*r* = .199, *p* = .001).

### 3.10 Mediation analyses

We designed two mediation models taking into account the results from correlational analyses. Particularly, we tested whether the relationships between attitude and generation of TMs and FMs were mediated by other variables.

In the first model, we entered the attitude as predictor, the FMC score as dependent variable, and the COPE‐avoiding strategies subscale and OK score as parallel mediators.

**FIGURE 2:**
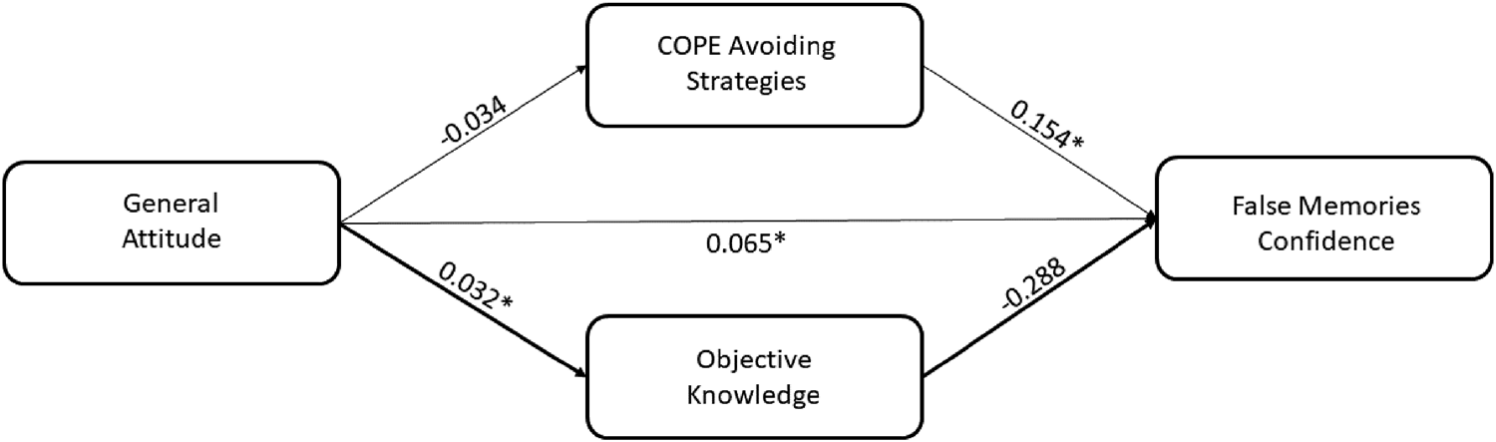
Scheme representing the mediation effect of objective knowledge in the relationship between general attitude and false memories confidence score controlling the effect of the other mediator (*p < .05)

We found that a more positive attitude was significantly related to a higher OK score (*B* = .032, *p* = .007) but not with COPE‐avoiding strategies (*B* = −.034, *p* = .346). Moreover, a higher FMC score was predicted by a larger use of avoiding strategies (*B* = .154, *p* = .001), whereas a trend toward significant was observed in the association between OK and FMC (*B* = −.288, *p* = .052). The 95% bias‐corrected CI based on 5000 bootstrap samples revealed that the indirect effect of attitude on FMC score through the OK score was statistically significant (estimate effect: −0.009; 95% CI: −.023, −.000), controlling the effects of other indirect effects (see Figure 2).

**FIGURE 3:**
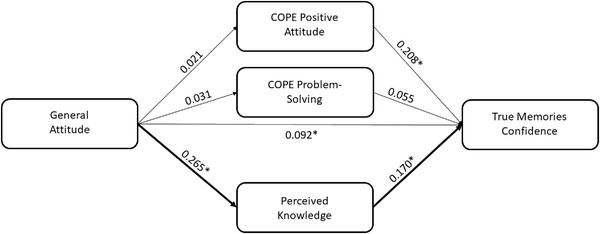
Scheme representing the mediation effect of perceived knowledge in the relationship between general attitude and true memories confidence score controlling the effect of the other mediators (*p < .05)

In the second model, the PK score and the COPE‐positive attitude and COPE‐problem‐solving subscales were entered as parallel mediators within the relationship between attitude and TMC score. We found a positive association between attitude and PK (*B* = .265, *p* < .001), whereas no significant associations with COPE‐positive attitude (*B* = .021, *p* = .587) and COPE‐problem‐solving (*B* = .031, *p* = .311) were detected. A higher TMC score was related to a larger use of positive attitude‐based coping strategies (*B* = .208, *p* = .004) and to a higher PK score (*B* = .170, *p* = .001) but not to COPE‐problem‐solving (*B* = .055, *p* = .539). The 95% bias‐corrected CI based on 5000 bootstrap samples revealed that the indirect effect of attitude on TMC score through PK was significant (estimate effect: 0.045; 95% CI: .016, .078), controlling the effects of other indirect effects (see Figure 3).

## 4 DISCUSSION

In the current study, we investigated the possible correlates of TMs and FMs about COVID‐19‐related news, with a special attention to the participants’ attitude (positive/negative) toward vaccines and GP. Results from our study bring out a picture that is difficult to interpret but still clearly highlight how attitude acts at different levels, playing a pivotal role in modulating individual behaviors, also in relation to health issues (Ajzen et al., 2018). For a general overview, the main findings emerged by analyzing the cross‐sectional data we collected are summarized below.

We found that respondents with negative attitude took anti‐SARS‐CoV‐2 vaccination later than those who showed a positive attitude. This might explain the highest number of reported infections by participants with negative attitude, appearing to be more susceptible to contract the virus than those with positive attitude.

Interestingly, respondents with positive attitude were more afraid that their loved ones contracted the COVID‐19 as compared with respondents showing negative attitude. This result is likely due to the lowest frequency of infections reported by participants with positive attitude. Indeed, one might speculate that the latter could have developed a higher health risk perception, that is, the fear of contracting the virus and then transmit it to their loved ones within the oldest age brackets, where the mortality rate is significantly higher (Calcaterra et al., 2021; Ilardi, Chieffi, Iavarone, et al., 2021; Ilardi, Chieffi, & Ilardi, 2021).

To acquire information about COVID‐19, participants with positive attitude reported using traditional media more than those with negative attitude; the opposite pattern was detected for social media. However, it is important to note that, among social media, respondents with positive attitude reported a greater use of scientific publishing channels, whereas participants with negative attitude accessed mainly social profiles of celebrities and family members or friends.

Finally, participants showing positive attitude got higher PK scores than those showing negative attitude.

Moving on to the central issues of TMs and FMs, participants reported higher confidence levels when required to judge their memory of true than fake news. As the former circulated through the media, whereas the latter were newly fabricated stories, this finding supports the robustness of our apparatus. However, participants with positive attitude were more confident about their memory of both true and fake news. Furthermore, although all participants showed a higher confidence for traditional media as compared with social media when asked to assess their source memory for both true and fake news, respondents with positive attitude reported the highest confidence levels. The differences observed between participants with positive and negative attitudes on memory testing may be ascribable to several factors.

TMC was found to be positively related to the fear of loved ones contracting the virus and negatively related to anxiety that is a well‐known factor potentially decreasing memory skills (Mueller, 1979; Storbeck et al., 2005). The enhancing effect of the fear of COVID‐19 supports the view that negative emotional valence may be a significant catalyzer of memory abilities, that is, it can improve encoding and maintenance of information, reducing reconstructive memory errors and thus the probability of generating FMs (Kaplan et al., 2015; Finucane, 2011). However, this result might be explained by an additional confounded variable, that is, the willingness to help, underlying a possible prosociality‐induced positive bias (Gaesser, 2013; Hikosaka, 2010; Itoi & Sugimoto, 2010; Jasmin et al., 2004; Kranz et al., 2010; La Marra et al., 2020; Scuotto et al., 2021; Villano et al., 2021). In other words, the fear that a loved one may become ill, suffer, or even die may lead to more carefully selecting the information disseminated about the disease, to filter them more thoroughly, and to construct more robust and durable mnestic traces in order to functionally cope with a potential emergency situation. When the goal is protecting others, people may be more motivated and committed, thus producing fewer cognitive errors (Scuotto et al., 2021).

The finding that respondents with positive attitude reported an increased fear that their loved ones contracted the COVID‐19 may explain the higher confidence levels for TMs as compared with participants showing negative attitude. This may also be the result of a larger use of traditional media to stay knowledgeable on the COVID‐19‐related arguments. Traditional media are preferred as a primary source because they are generally considered more authoritative, realistic, and truthful (Johnson & Kaye, 2015). In addition, when using social media, respondents with positive attitude often accessed scientific publishing channels; conversely, respondents with negative attitude typically consult less authoritative channels. Some social channels may promote the dissemination of fake news (Collins et al., 2021; Galeotti, 2019) influencing attitudes toward a given topic; specularly, an individual may unintentionally consult sources providing information that is consistent with her/his preexisting beliefs (confirmation bias; Lord et al., 1979).

Worthy of note is that participants with positive attitude were more confident in remembering both true and fake news, that is, they showed increased confidence even for FMs. This result may be due to the higher PK score. Indeed, a high PK is generally associated with an overestimation of memory skills, that is, the tendency to remember both true and false information more likely (Mehta et al., 2011).

Finally, we tested whether the relationships between attitude and TMs/FMs formation were mediated by the contributions of other variables. We found that attitude positively predicted confidence levels for both TMs and FMs; furthermore, the association between attitude and TMs was increased by PK, whereas OK was found to be a possible mediator in the relationship between attitude and FMs. More specifically, we found that a higher OK might dampen the positive association between attitude and FMC. These findings suggest that both PK and OK play pivotal roles in modulating memory abilities, with selective contributions that need to be investigated, in combination with attitude toward a given topic, in future research. Coping mechanisms, such as positive attitudes, problem‐solving, and avoidance strategies, although strictly related to memory skills (Zhu et al., 2010), were found not to be significant mediating variables.

The current study presents some limitations. The sample size is fairly small, and participants were young and highly educated. Still, although fake news were constructed after a careful examination of information spread by media, it is difficult to rule out the possibility that participants had already generated FMs for similar episodes/news. In this case, our experiment would have uncovered preexisting FMs rather than implanting them. Nevertheless, this scenario has been deemed quite unlikely. Lastly, one could assume that no substantial differences exist between the COVID‐19 OK quiz and the paradigm used to assess memory for true and fake news. In both cases, participants are presented with short sentences, differing mainly in terms of length, and they need to access to the content of their memory in order to give an answer, regardless of how it is delivered (false vs. true or remember vs. do not remember). However, OK score did not correlate with raw TM (*r* = .053, *p* = .373) and FM scores (*r* = −.102, *p* = .083). This result supports the independence of the two constructs. In fact, the OK questionnaire assessed—through a true/false format—the knowledge of certain notions related to COVID‐19, mainly requiring access to semantic memory. On the contrary, the misinformation paradigm was set up to determine whether participants remembered seeing/hearing COVID‐19‐related news, presented as in a newspaper headline, and most likely requiring access to episodic memory.

## AUTHOR CONTRIBUTIONS

Conceptualization, methodology, formal analysis, investigation, resources, data curation, writing original draft, review and editing, and visualization: Chiara Scuotto and Ciro Rosario Ilardi. Methodology, formal analysis, data curation, and writing original draft: Gianpaolo Maggi. Methodology, writing review, and editing: Alfonso Ilardi and Nadia Gamboz. Resources and visualization: Giovanni Borrelli. Project administration, supervision, methodology, writing review, and editing: Marco La Marra and Raffaella Perrella.

## CONFLICT OF INTEREST

The authors declare that they have no conflicts of interest.

### PEER REVIEW

The peer review history for this article is available at https://publons.com/publon/10.1002/brb3.2815

## Supporting information

Supporting Information

## Data Availability

Data analyzed during the current study are available from the corresponding author upon reasonable request.
