# Supplementary material for: What makes us more susceptible to false memories in the era of COVID‐19? A focus on vaccines and Green Pass
Source: Brain Behav. 2022 Nov 30;13(1):e2815. doi: 10.1002/brb3.2815 (PMC9847604; doi:10.1002/brb3.2815)
Supplement: Supplementary file 1 — Supporting Information [file BRB3-13-0-s001.docx]

**Appendix A**

**Attitude towards anti-SARS-CoV-2 vaccines and Green Pass**

1. The vaccine protects me from infection.
2. Getting vaccinated is good for the community.
3. The government reports accurate information about the effectiveness of vaccines.
4. The vaccine is safe.
5. The Green Pass is an effective tool for safeguarding my health.
6. The Green Pass is an effective tool for safeguarding the health of the community.
7. The Green Pass is a useful tool to revive the economy.
8. The Green Pass is a tool that protects personal freedoms.

**COVID-19 Perceived Knowledge (PK) questionnaire**

How do you assess your knowledge about […]

**COVID-19 (general)**

1. the differences between the various anti-SARS-CoV-2 diagnostic tests (for example, antigenic, molecular, serological)?

2. the statistical indices used to describe the impact of the pandemic and the consequences of the infection (for example, incidence, mortality, lethality)?

3. the differences between the Individual Protection Devices (DPI) commonly used, such as surgical masks, FFP2, and FFP3?

**Anti-SARS-CoV-2 vaccines**

4. the differences between the vaccines available in Italy in terms of mechanisms of action (e.g., mRNA vaccines, viral vector vaccines)?

5. the vaccine’s ability to protect from COVID-19?

6. the likelihood of occurrence of serious side effects (for example, pericarditis, myocarditis) induced by the administration of anti- oV-2 vaccines?

**Green Pass**

7. the Italian Government’s provisions on the Green Pass?

8. the differences between the basic Green Pass and the super Green Pass?

9. the provisions of the European Union regarding the validity of the digital COVID certificate?

**COVID-19 Objective Knowledge (OK) Quiz**

**COVID-19 (general)**

1. Wearing the surgical mask protects others more than oneself from contagion. (TRUE)

2. The R0 index expresses the ratio between individuals exposed to the virus who get sick and those exposed who do not get sick. (FALSE)

3. The serological test detects whether the individual has come into contact with the virus, but it is unable to confirm the presence of an infection in progress. (TRUE)

4. The molecular swab has a diagnostic sensitivity of approximately 60%. (FALSE)

**Anti-SARS-CoV-2 vaccines**

5. The first dose of vaccine begins to take effect 7-14 days after administration. (TRUE)

6. The international guidelines suggest not to consume food in the two hours prior to administration of the anti-SARS-CoV-2 vaccine. (FALSE)

7. Both the Moderna and the Pfizer-BioNTech vaccine are mRNA vaccines. (TRUE)

8. One in 1000 subjects has a high probability of developing myocarditis and pericarditis following vaccination with Pfizer-BioNTech or Moderna. (FALSE)

**Green Pass**

9. The first country to introduce the Green Pass was Belgium. (FALSE)

10. Basic Green Pass means the COVID-19 Green Certification issued following vaccination, recovery, or administration of antigenic/molecular test with negative result. (TRUE)

11. To ensure safety in travel between the member states of the European Union, the adoption of a digital COVID certificate has been proposed that exempts its holder from restrictions on free movement. (TRUE)

12. The super Green Pass is only issued following vaccination. (FALSE)

**Appendix B**

**True and Fake news**

**True News (Vaccines)**

TN1. The Health Minister, Roberto Speranza, has announced that from 1 December 2021 the third dose of the vaccine can be administered to all people between 40 and 60 years of age.

TN2. The Italian Society of Neonatology (SIN) and the Italian Society of Pediatrics (SIP) recommend vaccination against SARS-CoV-2/COVID-19 with mRNA vaccines for breastfeeding women, without the need to stop breastfeeding.

TN3. A study conducted by the University of Bristol and published in November 2021 verified that it is safe to administer the anti-COVID-19 vaccine and the flu vaccine in a single session.

**True News (Green Pass)**

TN4. Between September and October 2021, the Government of Denmark allowed access to nightclubs, restaurants, spas and stadiums even to subjects who did not have a Green Pass.

TN5. The Italian Government has established that from 10 January 2022 the super Green Pass is mandatory to access cultural, social and recreational centers that carry out outdoor activities.

TN6. In Italy, starting from 1 September 2021, the Green Pass has become mandatory to take advantage of long-distance transport, for example, airplanes, high-speed trains, Intercity, buses and ferries.

**Fake News (Vaccines)**

FN1. In line with the results of study by the Istituto Superiore della Sanità, the probability of becoming seriously ill because of SARS-CoV-2 infection following the third dose of the vaccine remains unchanged for people over the age of 80 due to the lower efficiency of their immune systems.

FN2. A study by San Raffaele Hospital (Milan) identified cases of otitis following the administration of the vaccine among 20% ​​of Italian children.

FN3. The AIFA Technical Scientific Commission (CTS), in its meeting on 1 December 2021, approved the use of the AstraZeneca vaccine for the age group 5-11 years, with a reduced dose and a specific formulation.

**Fake News (Green Pass)**

FN4. The French government, following the success of the vaccination campaign, decided that the possession of the Green Pass will no longer be necessary starting from February 2022.

FN5. In Switzerland, starting from January 2021, access to supermarkets is not allowed to people aged 40 or over who do not have the Green Pass.

FN6. The Italian Ministry of Health has established that, starting from December 2021, the possession of the super Green Pass, if becoming positive only based on the antigen test’s result, will allow you to avoid the quarantine period.
